# Improving compatibility between coffee or black tea ground wastes and polymer matrix via silane treatment for production sustainable biofillers

**DOI:** 10.1038/s41598-025-97980-7

**Published:** 2025-04-19

**Authors:** Bolesław Szadkowski, Magdalena Śliwka-Kaszyńska, Anna Marzec

**Affiliations:** 1 Institute of Polymer and Dye Technology, Faculty of Chemistry, Lodz University of Technology, Stefanowskiego 16, 90-537 Lodz, Poland; 2 Department of Organic Chemistry, Faculty of Chemistry, Gdansk University of Technology, Narutowicza 11/12, 80-233 Gdansk, Poland

**Keywords:** Bio-waste engineering, Coffee grounds, Tea grounds, Silane, Colorants, Polymer aging, Materials science, Biomaterials

## Abstract

The incorporation of bio-waste products into polymer materials as a fillers and colorants represents a highly significant approach for developing sustainable composites, aligning with the principles of a circular bioeconomy and contributing to reduced environmental impact. In this study, coffee grounds (CG) and black tea grounds (BTG), two mainstream food processing by-products, were employed as bio-fillers and pigments for ethylene-norbornene (EN) composites. The effects of hydrophobic treatment with (3-aminopropyl)triethoxysilane (APTS) on CG and BTG powders were compared to those of untreated bio-fillers with respect to dispersion, color characteristics, mechanical properties, and UV aging stability of the polymer composites subjected to 50, 100, 200 and 300 h of UV aging. Both the waste additives and the resulting composites were characterized through Fourier-transform infrared (FTIR) spectroscopy, tensile testing, thermogravimetric analysis (TGA), scanning electron microscopy (SEM) and spectrophotometric method. The obtained results demonstrated that the silanization of CG and BTG bio-fillers improved their dispersion within the EN matrix, consequently enhancing the UV aging resistance of the polymer composites. The EN/CG-APTS composite exhibited the best mechanical properties during the aging process, with the highest aging factor value of 0.6 after 300 h. On the other hand, the EN/BTG-APTS composite showed the smallest color change (ΔE = 7.8) after 300 h of aging. These findings indicate that improving the compatibility of bio-fillers with the polymer matrix can further increase their application potential in sustainable polymer materials technology.

## Introduction

In recent years, the pursuit of sustainable and environmentally friendly filler materials has driven both academic and industrial efforts to explore unconventional sources. These alternatives include bio-based fillers derived from renewable plant-based resources or recycled biowaste, such as natural fibers, wood flour, rice husks, and extracted cellulose^1–5^. These fillers not only help reduce material costs but also offer biodegradability while enhancing mechanical properties.

**Fig. 1:**
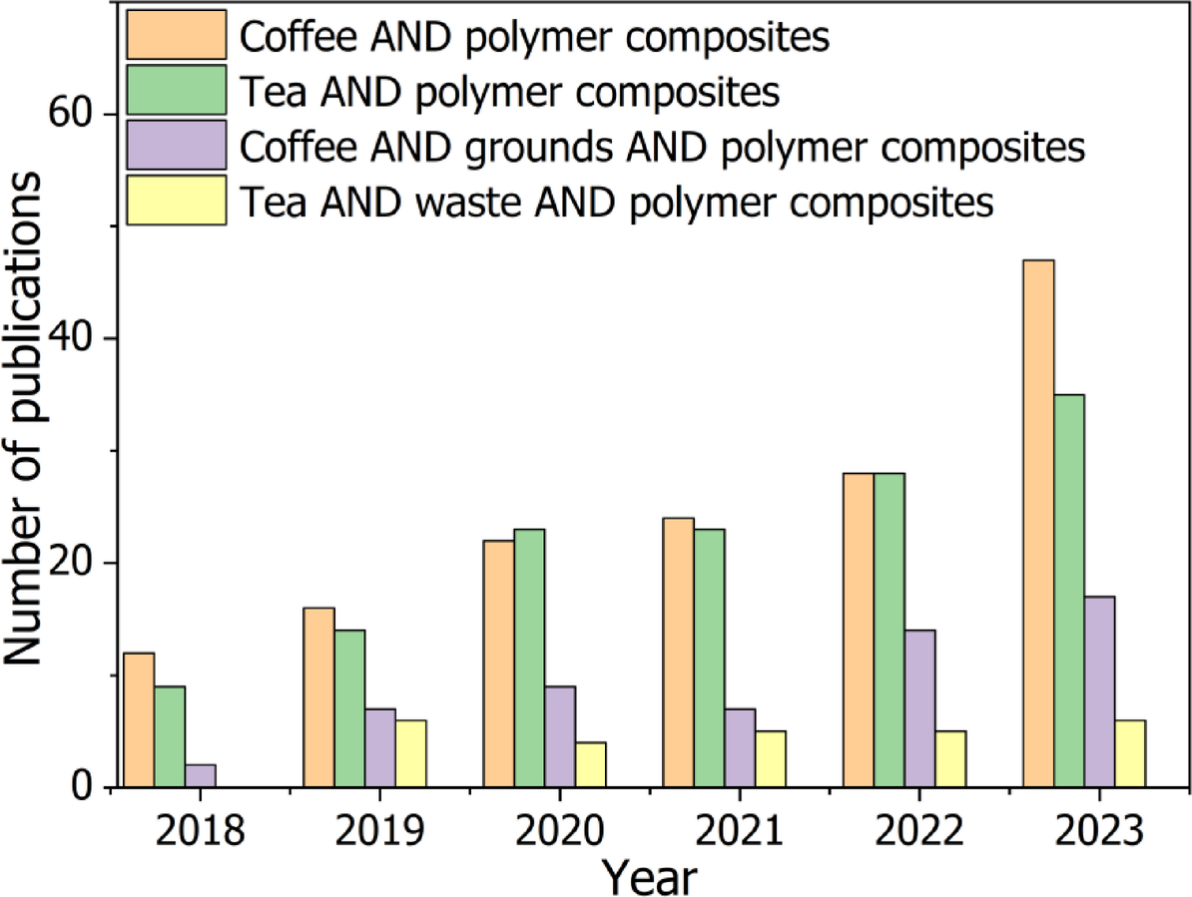
Number of publications found in the Scopus database for different combination terms related to coffee, tea and polymer composites.

Coffee and tea are among the world’s most widely consumed beverages, generating substantial waste during preparation, consumption, and disposal. Coffee has become an integral part of global culture, with annual coffee bean production in the 2021/2022 crop year surpassing 10 million metric tons—nearly double the amount produced 30 years prior^6^. As consequence, solid waste generated from coffee production amounts to nearly one million tons annually^6^. Meanwhile, global tea consumption is projected to reach approximately 7.4 million tons by the end of 2025^7^. Recently, there has been a surge in interest in various tea-based products, such as instant tea, tea-infused beverages, tea extracts, and tea seed oil. As a result, the substantial rise in tea consumption has led to a corresponding increase in tea waste. Population growth has further amplified demand, contributing to environmental challenges such as the inefficient management of organic waste and unsustainable resource use. However, owing to the presence of various bioactive compounds, including polyphenols, flavonoids, and catechins, these bio-fillers have the potential to alter properties of polymeric materials^8–10^. A review of the literature indicates that the use of tea and coffee waste in polymer composites is a relatively new topic, but it has been gaining increasing interest from researchers in recent years (Fig. 1). This surge in attention is driven by the increasing emphasis on sustainability and the potential of agro-waste to serve as cost-effective, renewable fillers in polymer matrices.

Both coffee and tea contain bioactive compounds that can affect the mechanical, thermal, and structural properties of various polymer matrices when incorporated as fillers^11^. One of the primary effects of these natural additives is on the mechanical performance of polymer composites. Coffee grounds, for instance, have been shown to improve the tensile and flexural strength of polyethylene composites^12^. This enhancement is attributed to the inherent rigidity and fibrous nature of coffee waste, which acts as a reinforcement within the polymer matrix. Moreover, coffee grounds can be used as biomass-derived charring agent to combine with flame retardants to form intumescent flame retardant (IFR) agent for polymer composites^13^. Vahabi et al.^14^ used raw and chemically modified spent coffee grounds to create an eco-friendly and highly effective flame retardant. The pyrolysis combustion flow calorimeter (PCFC) analysis demonstrated a significant decrease of 40% in the peak heat release rate (pHRR). Coffee grounds has been also reported as renewable waste material improving the biodegradation properties of polymer composites, contributing to the circular economy. The literature demonstrates that higher loadings of this bio-filler significantly accelerate biodegradation while maintaining adequate structural performance, positioning poly(butylene succinate) (PBS) bio-composites as promising materials for sustainable applications^15^.

Tea waste, on the other hand, can similarly enhance polymer composites^16^. Rich in lignocellulosic material, tea leaves can improve the stiffness and strength of polymer composites^16^. Tea grounds have been also reported as materials that can affect the cross-linking and tensile properties of elastomers. It was showed that incorporation of 10 phr of ground tea waste particles into natural rubber (NR) matrix leads to increase tensile strength by about 4.4 MPa when compared to the raw NR^17^. Moreover, the antioxidants in tea may confer added resistance to UV radiation, further improving the durability of polymer-based materials^18^. Very recently, coffee and tea bio-fillers were used as hybrid systems with common fillers such as montmorillonite (MMT), silica (SiO_2_), and cellulose to improve UV-aging resistance of ethylene–norbornene copolymer (EN)^19^. The authors observed that the application of 20 phr of hybrid fillers enhanced UV resistance of EN, as evidenced by high aging factor parameters and relatively low color changes of the materials after 400 h of UV exposure.

However, both coffee and tea waste additives can also introduce challenges. One significant issue is the poor interfacial adhesion between the hydrophilic natural fillers and hydrophobic polymer matrices, which can lead to reduced mechanical performance if not properly addressed. Hejna et al.^20^ have noticed that increasing concentration of coffee and cocoa by-products by more than 5 wt% resulted in significant reduction in tensile strength and elongation at break of polyethylene composites. Therefore, challenges such as filler-matrix adhesion need to be addressed to optimize the performance of agro-waste fillers in industrial applications. To mitigate this, surface treatments or compatibilizers can be proposed to enhance bonding between the filler and the polymer^21–25^. To the best of our knowledge, limited attention has been devoted to the compatibilization of black tea or coffee ground waste bio-fillers with a polymer matrix via silane treatment to achieve uniformly colored polymer composites with enhanced functional properties and improved resistance to UV radiation.

**Fig. 2:**
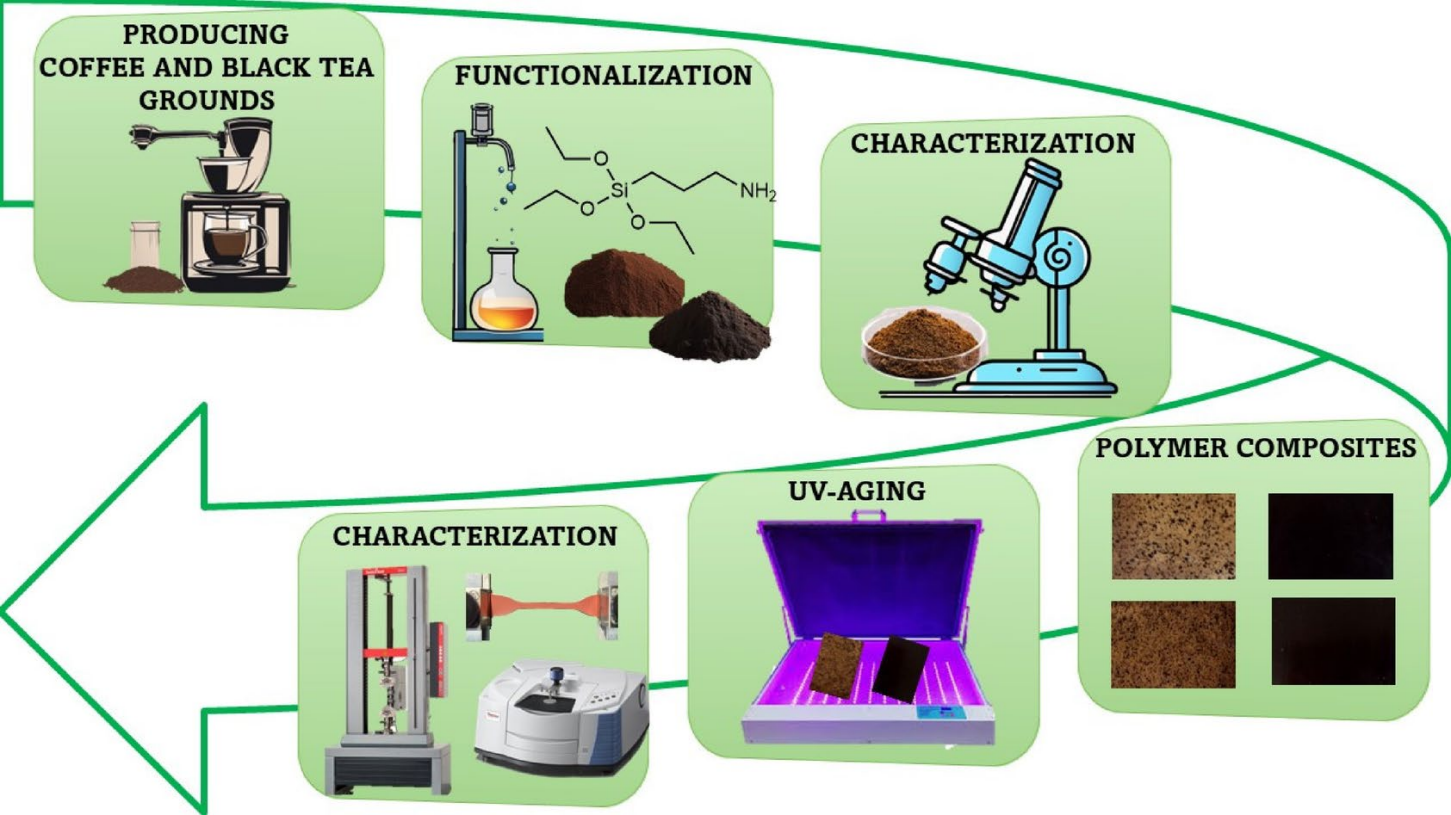
Schematic illustration of the research concept.

The aim of this work is to determine the potential of waste coffee and tea grounds as bio-fillers and coloring substances in polymer materials. In this context, the current studies focuses on the enhancement the compatibility between agro-waste fillers, such as coffee and tea grounds, and an EN copolymer matrix through the silanization process using 3-aminopropyltriethoxysilane (APTS) as a coupling agent. The silanization aims to improve the interfacial bonding between the hydrophilic bio-fillers and the hydrophobic polymer matrix, addressing challenges related to poor adhesion. The presence of tea and coffee grounds in the polymer matrix may contribute to enhancing the polymer’s resistance to unfavorable aging conditions. Therefore, this study investigates the influence of both raw and silanized fillers on the surface and mechanical properties of the resulting composites, with a focus on understanding how the presence of silane-modified bio-fillers protects the polymer matrix against long-term UV exposure (Fig. 2). UV aging behavior was analyzed using Fourier Transform Infrared Spectroscopy (FTIR), mechanical testing, and colorimetric methods.

## Experimental

### Materials

Ethylene-norbornene random copolymer (EN) with a tradename of Topas Elastomer 140 (40 wt% bound norbornene content) was purchased from Topas Advanced Polymers (Germany). Coffee grounds (CG) and black tea grounds (BTG) were collected from local sources and used as raw materials. 3-aminopropyltriethoxysilane (APTS) was obtained from Sigma-Aldrich (Germany) and used without purification. Ethyl alcohol (95%) and toluene (96%) were purchased from Sigma-Aldrich (Germany), and deionized water was used for washing the bio-fillers. Methanol (MeOH) and acetonitrile (ACN) of HPLC grade were purchased from Merck (Darmstadt, Germany). Formic acid (FA, 98–100%) was purchased from Fisher Chemical (Hampton, USA). All aqueous solutions were prepared using Milli Q water.

### Extraction procedure

Ground coffee and black tea (~ 4 g) were suspended in boiling water (50 mL) and stirred for 30 min until cooled. The mixtures were centrifuged at 9000 rpm for 5 min to separate the particulate matter. Supernatants were then filtered over a 0.45 μm RC syringe filter.

### LC-MS analysis

2 µL of the samples were injected onto RP-octadecyl column (Poroshell EC; 2.7 μm, 3.0 × 150 mm, Agilent Technologies) thermostated at 40 °C. The elution was performed using 0.1% (v/v) formic acid in water (solvent A) and ACN/MeOH (1:1; v/v) (solvent B) in gradient mode as follows: 0–15 min 10% B; 30 min 50% B; 40 min 100% B; 45 min 100% B for both extracts. The mobile phase flow rate was 0.4 mL min^− 1^. The UV signal was registered at 254, 280 and 350 nm. All mass-spectrometric scan data (m/z 50–1500) were recorded in negative and positive ionization scan modes. The nebulizer pressure, nitrogen flow rate, drying gas temperature, drying gas flow rate, and sheath gas temperature were 45 psi, 5 L min^− 1^, 300 °C, 11 L min^− 1^ and 250 °C, respectively. The capillary voltage was 3.5 kV and the fragmentation voltages was 150 V.

Chromatographic analysis was performed using Agilent liquid chromatograph series 1290 (Agilent Technology, Waldbronn, Germany) consisting of binary pump G4220A, autosampler G4226A, thermostated column compartment G1316C, diode-array detector G1315C, and triple quadrupole mass spectrometer G6460 with AJS electrospray ionization source. The structures of the identified compounds were confirmed by HPLC-ESI(-)-QTOF analysis using an Agilent 1290 LC system coupled to the Agilent QTOF mass spectrometer G6546A (Santa Clara, CA, USA). The chromatographic system was controlled with Agilent MassHunter software B 06.01.

### Silanization

The silanization process was conducted as follows: coffee and tea grounds were initially dried at 80 °C for 24 h to eliminate moisture. Subsequently, 50 g of the dried grounds were immersed in a solution of 5% (w/v) APTS in toluene for 24 h at room temperature. After the immersion period, the mixture was filtered, and the silanized coffee grounds (CG-APTS) and tea grounds (BTG-APTS) were thoroughly washed with ethanol. The bio-fillers were then dried again at 80 °C and stored in a desiccator until further use.

### Preparation of EN composites with coffee and tea grounds

Polymer composites were prepared in a Brabender N50 measuring mixer (Duisburg, Germany) at 110 °C with a rotor speed of 50 rpm for 30 min. Due to the fact that the CG and BTG used in this study were considered as intermediate additives between natural pigments and bio-fillers, they were incorporated into the polymer matrix at a concentration of 5 wt%. The formulations for the pre-pared EN composites were as follows (where phr means parts per hundred parts of polymer):

- 100 phr of EN copolymer,
- 100 phr of EN copolymer filled with 5 phr of raw coffee grounds (EN/CG),
- 100 phr of EN copolymer filled with 5 phr of raw black tea grounds (EN/BTG),
- 100 phr of EN copolymer filled with 5 phr of silanized coffee grounds (EN/CG-APTS),
- 100 phr of EN copolymer filled with 5 phr of silanized black tea grounds (EN/BTG-APTS).

After blending, the polymer composites were pressed between two steel plates under 15 MPa pressure at 120 °C for 5 min in a heated hydraulic press (Skamet 54436, SKA-MET, Skarzysko-Kamienna, Poland) to obtain the final plate-shaped samples.

### Characterization

The particle size distribution of the raw and silanized coffee and tea grounds was determined using a set of standard sieves with mesh sizes ranging from 63 μm to 250 μm. The ground material was sieved with a vibratory sieve shaker ANALYSETTE 3 PRO, Fritsch (Idar-Oberstein, Germany). Approximately 50 g of each sample was placed on the top sieve, and the stack was vibrated for 10 min using a sieve shaker. The weight% retained on each sieve was recorded.

The infrared absorption spectra were measured using a Thermo Scientific Nicolet 6700 FTIR spectrometer (Thermo Fisher Scientific, Waltham, MA, USA). The FTIR method was employed to analyze the composite samples within the wavelength range of 4000–500 cm^−1^ to investigate the formation of oxidation products during aging process. Variations in the relative absorption intensity of the ketone group –C =O (corresponding to 1800–1680 cm^−1^) compared to the absorption intensity of the methylene group –CH_2_– (at 3000–2800 cm^−1^) were used to determine the carbonyl index (CI) according to Eq. (1)^26^:

1 $$\:CI=\frac{{A}_{C=O}}{{A}_{-CH2-}},$$

SEM was performed using a LEO 1530 Gemini scanning electron microscope (Zeiss/LEO, Oberkochen, Germany) to examine the surface morphology of raw and silanized biofillers. Samples were sputter-coated with gold to prevent charging and enhance image quality.

The thermal stability of the samples was investigated using a Mettler Toledo Thermogravimetric Analyzer TGA/DSC1 (Mettler Toledo, Greifensee, Switzerland). Powder samples of approximately 10 mg were placed in an aluminum oxide crucible and heated from 25  to 600 °C in an argon atmosphere, with a heating rate of 10 °C/min.

Color measurements were taken using a Konica Minolta CM-3600d spectrophotometer (Konica Minolta Sensing, Inc., Osaka, Japan) in a spectral range from 360 to 740 nm. The total color change (ΔE) was calculated based on the CIE Lab* color space before and after UV aging. The ΔE of the samples was determined based on the following Eq. (2)^27^:

2 $$\:\varDelta\:E=\sqrt{\left({\varDelta\:a}^{2}\right)+\left({\varDelta\:b}^{2}\right)+\left({\varDelta\:L}^{2}\right)},$$

where ΔL is the level of lightness or darkness, Δa is the relationship between redness and greenness, and Δb is the relationship between blueness and yellowness.

A universal testing machine (Zwick/Roell 1435) (Zwick Roell Group, Ulm, Germany) was used to measure the tensile properties of the EN composites at a uniform crosshead speed of 500 mm/min, according to the ISO 37 standard. The results for tensile strength were recorded as the averages of five tests. The aging factor (AF) was calculated as the numerical change in the mechanical properties of the samples upon aging (3)^28^:

3 $$\:AF=\frac{{\left({T}_{S}\cdot\:{E}_{B}\right)}_{after\:aging}}{{\left({T}_{S}\cdot\:{E}_{B}\right)}_{before\:aging}},$$

where T_S_ is the tensile strength and E_B_ is the elongation at break of the samples.

Accelerated UV aging was performed in an Atlas UV 2000 apparatus. The procedure included two successively repeating segments:

- Day segment (irradiance 0.7 W/m2, temperature 60 °C, duration 8 h);
- Night segment (no UV radiation, temperature 50 °C, duration 4 h).

The progress of aging of samples was monitored after 50 h, 100 h, 200 h, and 300 h of the experiment.

## Results and discussion

### Coffee and tea grounds characterization

**Fig. 3:**
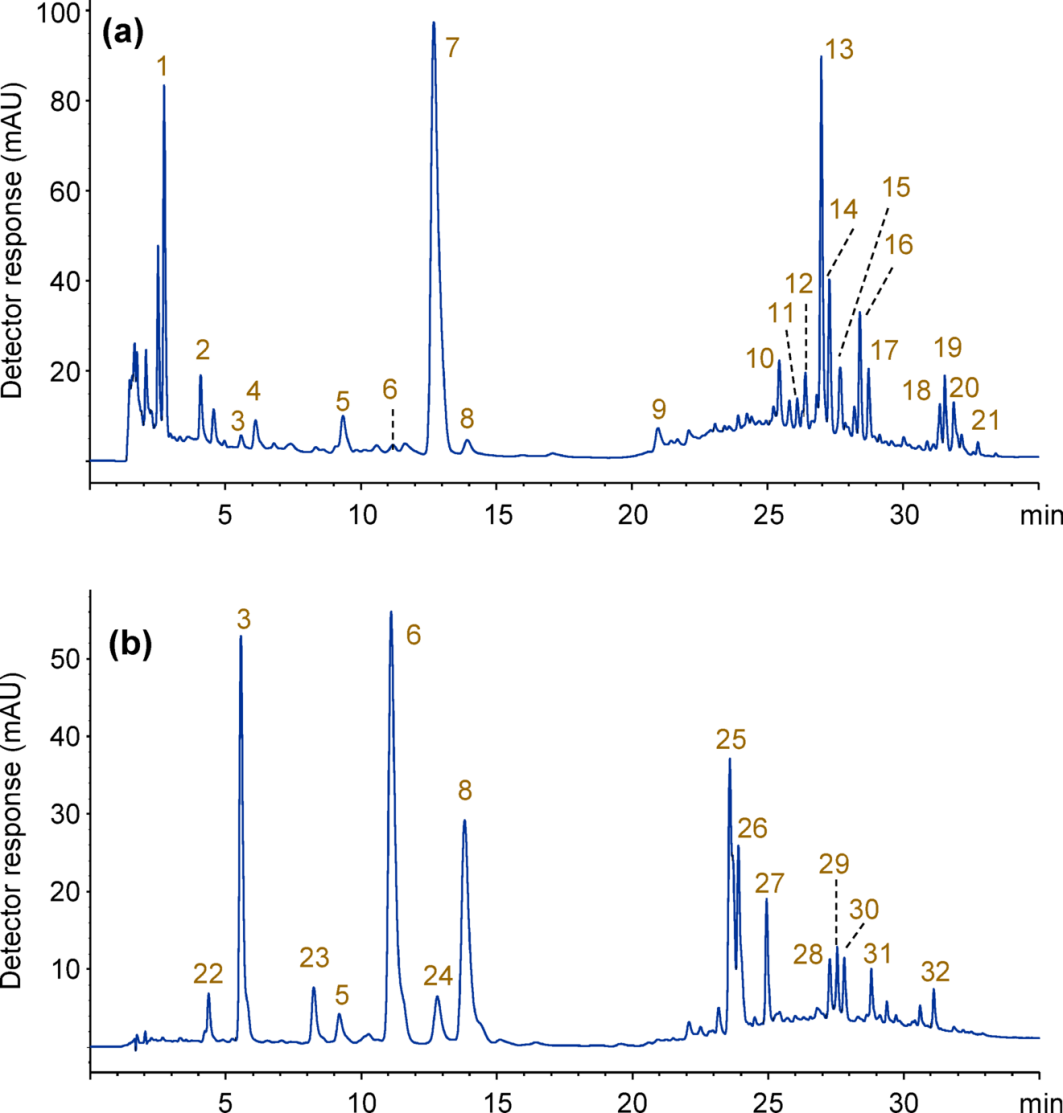
Chromatograms of extracts taken from: (**a**) a black tea and (**b**) ground coffee. For chromatographic conditions, see experimental section. List of major compounds corresponding to the labelled peaks are shown in Table 1.

LC-MS/MS approaches have been used to identify compounds present in a black tea and ground coffee water extracts. Figure 3 shows the chromatograms obtained for these extracts. The major peaks detected in each chromatogram are labelled with peak numbers ranging from 1 to 21 for black tea and from 22 to 32 for ground coffee. Compounds corresponding to each peak were tentatively identified based on their retention times, UV, and mass spectra in both negative and positive ionization modes (ESI(-/+) MS and MS/MS), in comparison with those reported in the literature. The retention times, molecular ions, main fragment ions, and proposed formulae for the compounds are summarized in Table 1.

These compounds represent a diverse class of phytochemicals, including organic acids, phenolic acids, chlorogenic acids, and flavonoids in the coffee extract. In addition, the tea extract contains catechins, teaflavins, and purine alkaloids^29,30^. Gallic acid (peak 1) is the predominant phenolic acid observed in black tea extracts (Fig. 3a). The tea sample also contains a significant amount of caffeine (peak 7). The major catechins identified are epigallocatechin, epicatechin, and epicatechin gallate (peaks 9, 10, and 13). Minor compounds detected in the black tea extract include caffeoyl- and coumaroylquinic acids, as well as glycoside forms of kaempferol and quercetin. Small amounts of teaflavins (peaks 18–21) were also found. The concentrations of these compounds are related to the quality of the tea leaves and the degree of fermentation during tea processing^31–33^. The most abundant group of compounds found in ground coffee are chlorogenic acids: 3-caffeoylquinic acid, 5-caffeoylquinic acid, and 4-caffeoylquinic acid, labelled as peak numbers 3, 6, and 8, respectively, as well as feruloylquinic acids, labelled as peaks 23, 24, 25, 26, and 27 in Fig. 3b. Other minor compounds tentatively identified in the negative ion mode in the coffee sample include dicaffeoyl- and coumaroylquinic acids, as well as caffeoyltryptophan (Table 1). These results are consistent with data found in the literature^34–36^.

**Table 1:** Spectrochromatographic data of the identified compounds.

Peak no.	t_*R*_ (min)	[M-H]^-^ (m/z)	[M + H]^+^ (m/z)	Fragment ions (m/z)	Proposed identification
1	2.7	169.0	–	151, 125, 70	Gallic acid
2	4.1	761.1	–	591, 465, 169	Theasinensin B
3	5.5	353.1	-	191, 161	3-Caffeoylquinic acid
4	6.1	759.1	–	607, 427, 337	Theacitrin A
5	9.2	337.1	–	191, 163, 119	3-Coumaroylquinic acid
6	11.1	353.1	–	191	5-Caffeoylquinic acid
7	12.7	-	195.1	138	Caffeine
8	13.8	353.1	–	289, 191, 173, 135	4-Caffeoylquinic acid
9	20.9	289.1	–	245, 203, 151	Epicatechin
10	25.4	457.1	–	169, 125	Epigallocatechin gallate
11	25.8	593.2	–	367, 285	Isovitexin-7-O- glucopyranose
12	26.1	337.1	–	273, 173, 163, 119	5-Coumaroylquinic acid
13	26.9	441.1	–	289, 245, 203, 169	Epicatechin gallate
14	27.3	771.2	–	609, 447, 301, 300	Quercetin- glucose-rhamnose-glucose
15	27.7	609.2	–	463, 291	Quercetin-3-rutinoside
16	28.4	739.2	–	593, 431, 285	Kaempferol-rhamnose-glucose-rhamnose
17	28.7	447.1	–	284, 255, 227	Kaempferol-3-glucopyranoside
18	31.3	–	565.1	139	Theaflavin
19	31.5	–	717.2	139	Theaflavin-3-gallate
20	31.9	–	717.1	139	Theaflavin-3′-gallate
21	32.7	–	869.2	717, 565, 139	Theaflavin-3,3′-digallate
22	4.4	153.0	–	123, 109	Protocatechuic acid
23	8.2	367.1	–	261, 209, 145	1-Feruloylquinic acid
24	12.8	367.1	–	261, 145	3-Feruloylquinic acid
25	23.6	367.1	–	253, 145	5-Feruloylquinic acid
26	23.9	367.1	–	337, 145	4-Feruloylquinic acid
27	24.9	735.2	–	465, 367	4-Feruloylquinic acid dimer
28	27.3	515.1	–	353, 259, 193	3,5-Dicaffeoylquinic acid
29	27.5	515.1	–	353, 191, 179	3,4-Dicaffeoylquinic acid
30	27.8	515.1	–	353, 191, 179, 135	4,5-Dicaffeoylquinic acid
31	28.8	365.1	–	229, 203, 186	Caffeoyltryptophan
32	31.1	699.3	–	349, 305, 186	Coumaroyltryptophan dimer

**Fig. 4:**
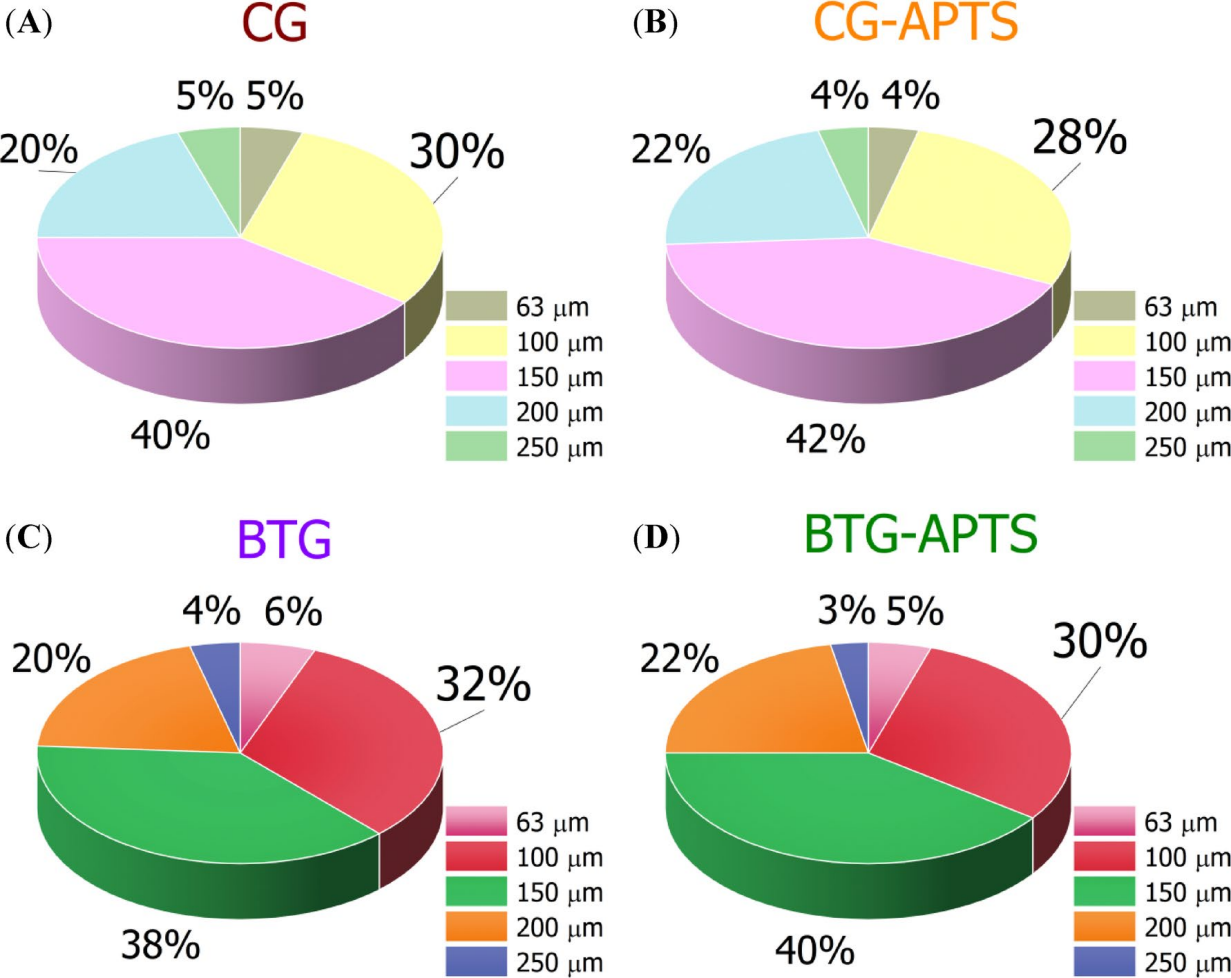
Particle size distribution of coffee grounds (**A**), silanized coffee grounds (**B**), black tea grounds (**C**) and silanized black tea grounds (**D**).

At the initial stage of the work, a grinding procedure was performed, which converted the solid grounds of coffee and black tea into a fine powder, significantly reducing the particle size. The cumulative distributions obtained using sieve analysis have been depicted in Fig. 4. As shown in Fig. 4, the largest volume fraction of all the bio-fillers, both before and after silane treatment, consists of particles with a size equal or lower than 150 μm, with percentage content ranging from 38% for BTG to 42% for CG-APTS. These results differ slightly from those obtained by Marques et al.^23^, who reported the largest fraction of spent coffee grounds particles with a size of 300 μm. However, it should be noted that both the particle size and size distribution of bio-waste fillers are strongly influenced by their source and the preceding milling process of the coffee grounds which can explain this differences. For both CG and BTG, the silanization process resulted in a slight increase (by 2%) in the volume fraction of particles with a size of 150 μm. Therefore, due to the predominant volume fraction, filler particles with sizes up to 150 μm were used as new bio-additives for the EN composites.

**Fig. 5:**
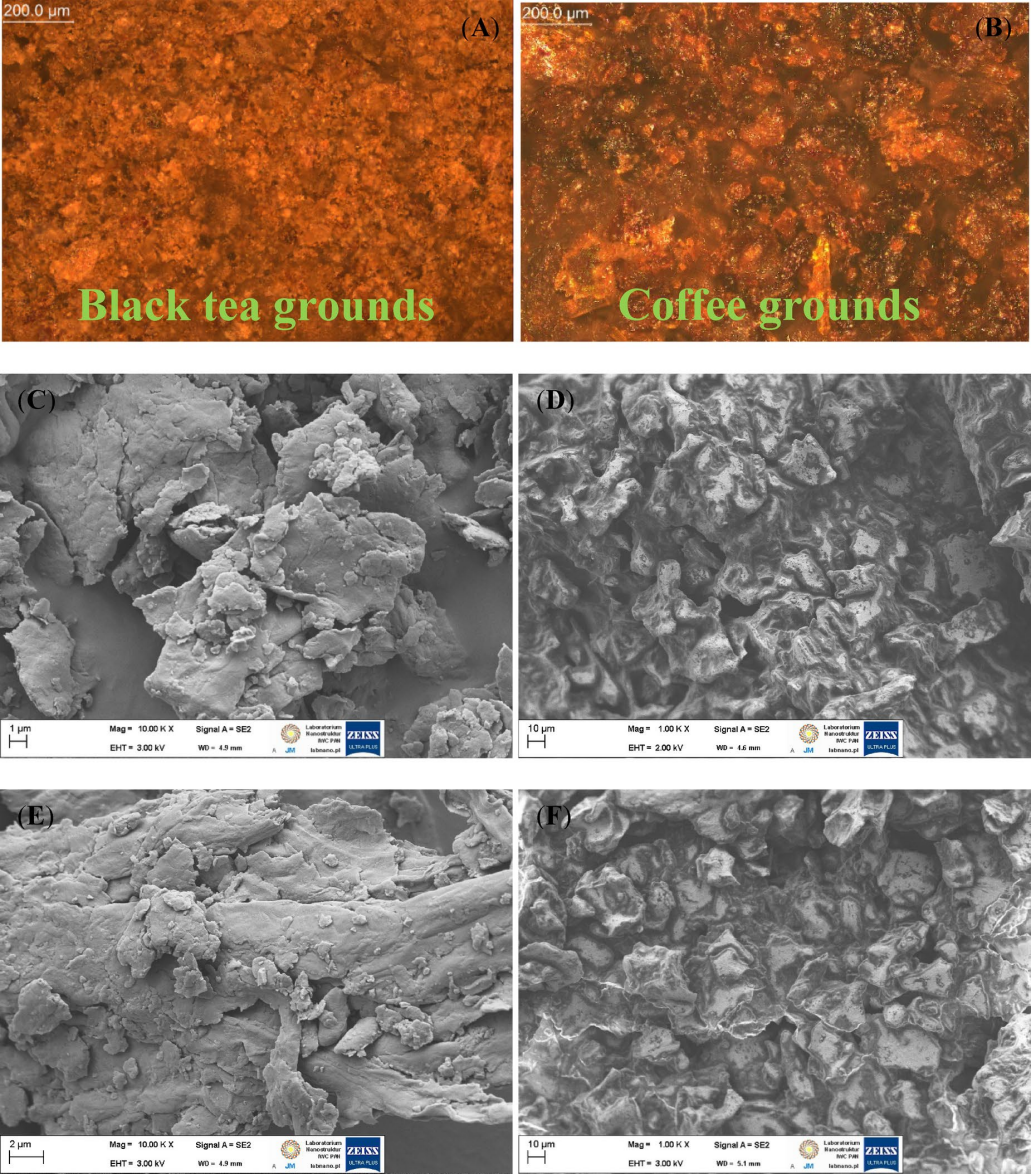
Optical and scanning electron microscopy images of black tea (**A**, **C**, **E**) and coffee (**B**, **D**, **F**) grounds.

Surface morphology of coffee and black tea grounds variants were examined by SEM and presented in Fig. 5. SEM analysis revealed that CG particles were denser, more irregular, erratic, and abrasive in shape compared to BTG particles. Rizal et al.^37^ have also investigated of the morphology of CG used as bio-filler for biopolymer packaging materials. They stated that the rougher surface of the bio-filler might lower the polymer’s ability to wet the fillers. The enhanced surface of the particles engages with the filler’s contact area within the matrix, improving mechanical adhesion on a more stable surface. The effect of silane treatment is most evident in black tea grounds. Raw BTG showed rougher, more irregular surfaces with visible larger agglomerates, whereas BTG-APTS exhibited smoother surfaces with a more compact structure, indicating successful surface modification. The higher effectiveness of surface modification for BTG compared to CG can be attributed to differences in their chemical composition and structural characteristics. Black tea grounds contain a higher proportion of polyphenolic compounds and lignin, which provide more reactive hydroxyl (–OH) groups for silane coupling reactions^35,38^. Additionally, the cell wall structure of tea grounds may facilitate better penetration and attachment of the silane molecules, enhancing the overall modification efficiency. In contrast, coffee grounds contain more residual oils, which may partially hinder the interaction between the silane agent and the filler surface, reducing the extent of modification.

**Fig. 6:**
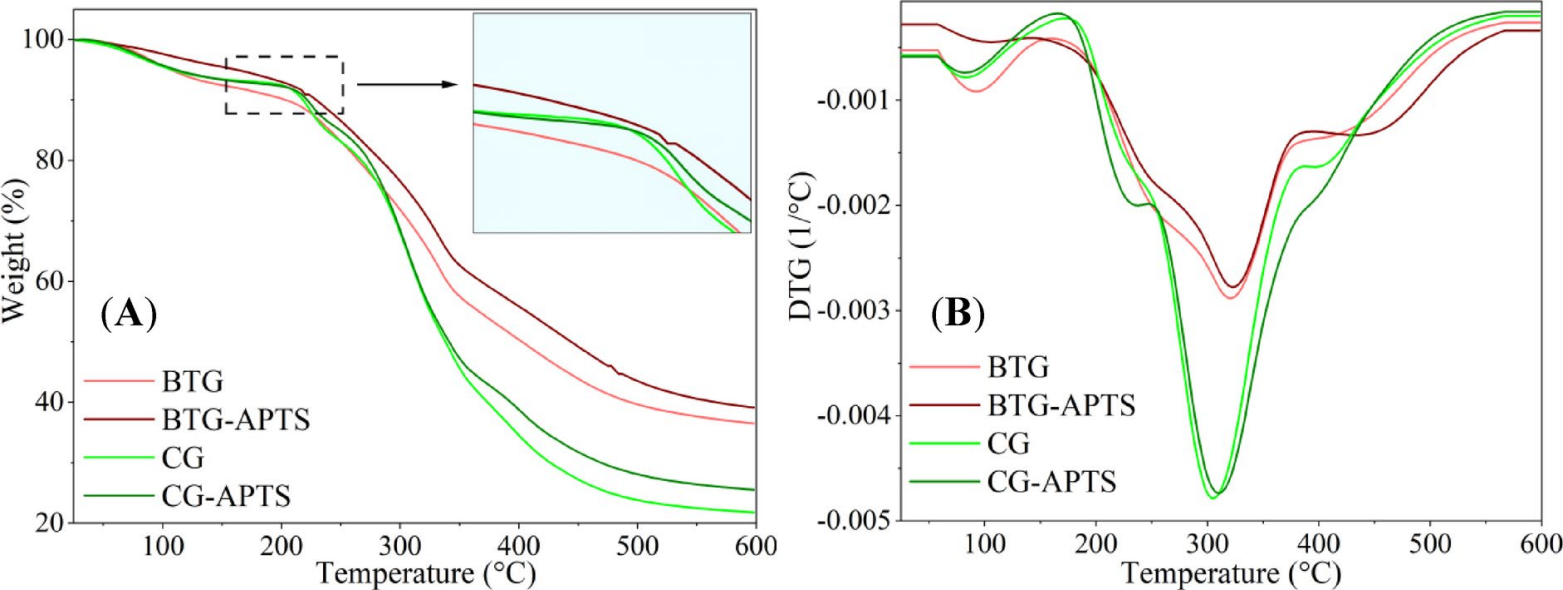
TGA profiles (**A**) and DTG curves (**B**) of the CG and BTG bio-fillers before and after silane treatment.

Thermal decomposition behavior of the CG and BTG before and after APTS silane treatment was investigated using thermogravimetric analysis (TGA). TGA/DTG profiles and corresponding thermal decomposition data of the studied materials are presented in Fig. 6; Table 2. The thermal decomposition of CG and CG-APTS proceeded through three distinct stages. In the first stage, a minor peak, centered around 75 °C and occurring between 30 °C and 150 °C, was observed, corresponding to the evaporation of adsorbed moisture in the sample^39^. The DTG analysis showed the most prominent peaks at around 300 °C, which can be attributed to the decomposition of hemicellulose in the coffee grounds, contributing nearly half of the sample’s dry weight. The third decomposition stage, occurring above 350 °C, was associated with the degradation of cellulose and lignin in the CG and CG-APTS compounds. For BTG and BTG-APTS samples, the thermal response in TGA measurements can be clearly divided into three main decomposition events. Similar to the case of CG, the first stage, corresponding to approximately 10% mass loss within the temperature range of 60–150 °C, was associated with the removal of physically bound water. In the second stage of decomposition, occurring between 200 °C and 550 °C, approximately 60% of the total mass is lost. This is primarily due to the release of volatile compounds from organic materials, including hemicellulose, cellulose, lignin, and other macromolecules^40^. Above 550 °C, the remaining components of BTG, such as char and minerals, underwent slow decomposition^41^. Importantly, the silanization process enhanced the thermal stability of the bio-fillers under investigation. The TGA data included in Fig. 6 revealed that the CG-APTS sample exhibited shifts in T_5_, T_10_ and T_20_ by 3 °C, 3 °C, and 6 °C respectively, compared to CG. More evidently, BTG-APTS demonstrated shifts of 56 °C, 27 °C and 18 °C, respectively, relative to raw BTG. Moreover, silane treatment of CG and BTG resulted in slight increase in the mass of charred residue. The TGA/DTG results indicate that the silanization of CG and BTG with APTS improves their thermal stability and may enhance their potential for application in polymeric materials. Scientific literature suggests that silanization can enhance the thermal stability of such bio-fillers. For instance, García-García et al.^42^ investigated the influence of silanization using (3-glycidyloxypropyl) trimethoxysilane (GLYMO) on coffee ground powder incorporated into polypropylene (PP) composites. Their findings demonstrated that the inclusion of silane-modified CG resulted in an enhancement of thermal stability by over 8% compared to non-functionalized counterparts. Furthermore, research conducted by Hayeemasae et al.^43^ assessed the utilization of silane-treated tea waste powder as a reinforcing agent in natural rubber composites. The study revealed that silane modification contributed to improved thermal stability, as confirmed by superior performance in thermal aging tests carried out at 100 °C over a 48-hour period. Indeed, the enhancement of the thermal stability of bio-fillers after silane treatment can be attributed to multiple interrelated mechanisms. This treatment effectively reduces the hydrophilicity of the bio-filler, minimizing moisture absorption, which is a known factor contributing to premature thermal degradation. Furthermore, the silane molecules form a stable siloxane network through hydrolysis and condensation reactions, which strengthens the filler structure and increases its resistance to thermal decomposition. This siloxane layer may also act as a barrier, restricting volatile degradation byproducts from escaping, thereby delaying the onset of thermal degradation.

**Table 2:** Thermal properties of CG and BTG bio-fillers determined in TGA measurements.

Sample	T_05_ (°C)	T_10_ (°C)	T_20_ (°C)	T_50_ (°C)	Char residue 600 °C (%)
BTG	106	202	265	403	36
BTG-APTS	162	229	283	445	39
CG	109	217	268	340	22
CG-APTS	112	220	274	340	26

T_05,10,20,50_— decomposition temperatures: 5%,10%,20% and 50% of sample mass.

### Application of bio-fillers in polymer materials

The APTS silane treatment chemically modifies coffee grounds and black tea grounds by introducing amine-functionalized silane groups onto the surface of the bio-fillers. This process occurs through hydrolysis of the ethoxy groups in APTS, forming reactive silanol groups that can form covalent bonds or hydrogen bonds with hydroxyl groups present on the lignocellulosic structure of coffee and tea waste. Coffee grounds and black tea grounds are lignocellulosic materials composed primarily of cellulose, hemicellulose, and lignin, along with proteins, polyphenols, and residual oils. The presence of hydroxyl (-OH) and other polar functional groups in these biopolymers provides reactive sites for chemical modification, such as silanization, enabling improved compatibility with hydrophobic polymer matrices. Therefore, this modification may enhance the interfacial adhesion between the bio-fillers and the polymer matrix, improving dispersion and mechanical properties of the composite.

**Fig. 7:**
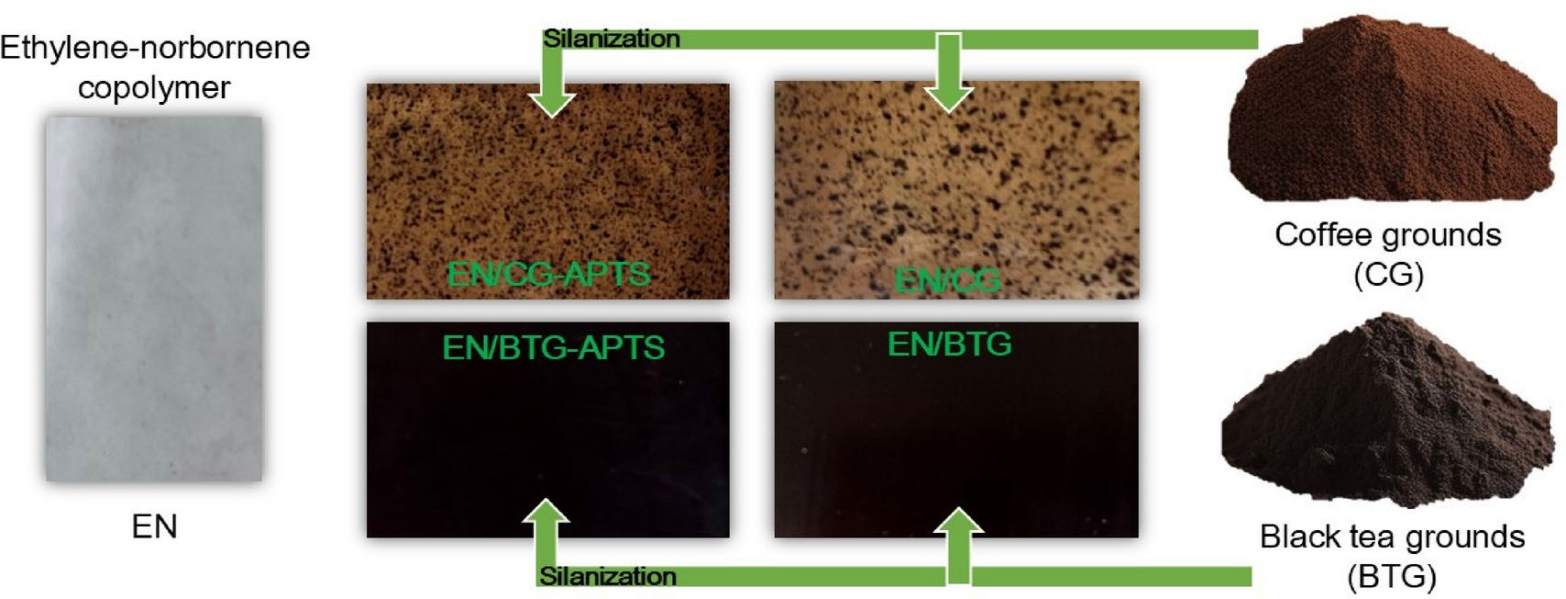
Digital images of the EN composites filled with raw and silanized CG and BTG bio-fillers (images taken by the corresponding author).

The application potential of CG and BTG and the influence of the silanization process on improving their compatibility with the polymer matrix were investigated in an EN copolymer, which is a transparent polymer that can be used in the packaging industry. CG, BTG, and their silanized forms were introduced as novel bio-fillers into EN composites at a concentration of 5 wt%, followed by exposure to UV aging for 50, 100, 200, and 300 h. As shown in Fig. 7, the addition of 5 wt% BTG imparted a uniform and intense coloration to the EN composite, while CG was poorly dispersed, with numerous visible agglomerates observable to the naked eye. Interestingly, the silanization process improved their coloring efficiency by achieving more uniform dispersion within the EN matrix. The digital image of the EN/CG-APTS composite shows significantly better dispersion of bio-filler particles, forming smaller agglomerates. The compatibilization effect achieved using a silane coupling agent may enhance not only the aesthetic qualities of the polymer composite but also its mechanical properties and aging resistance.

**Fig. 8:**
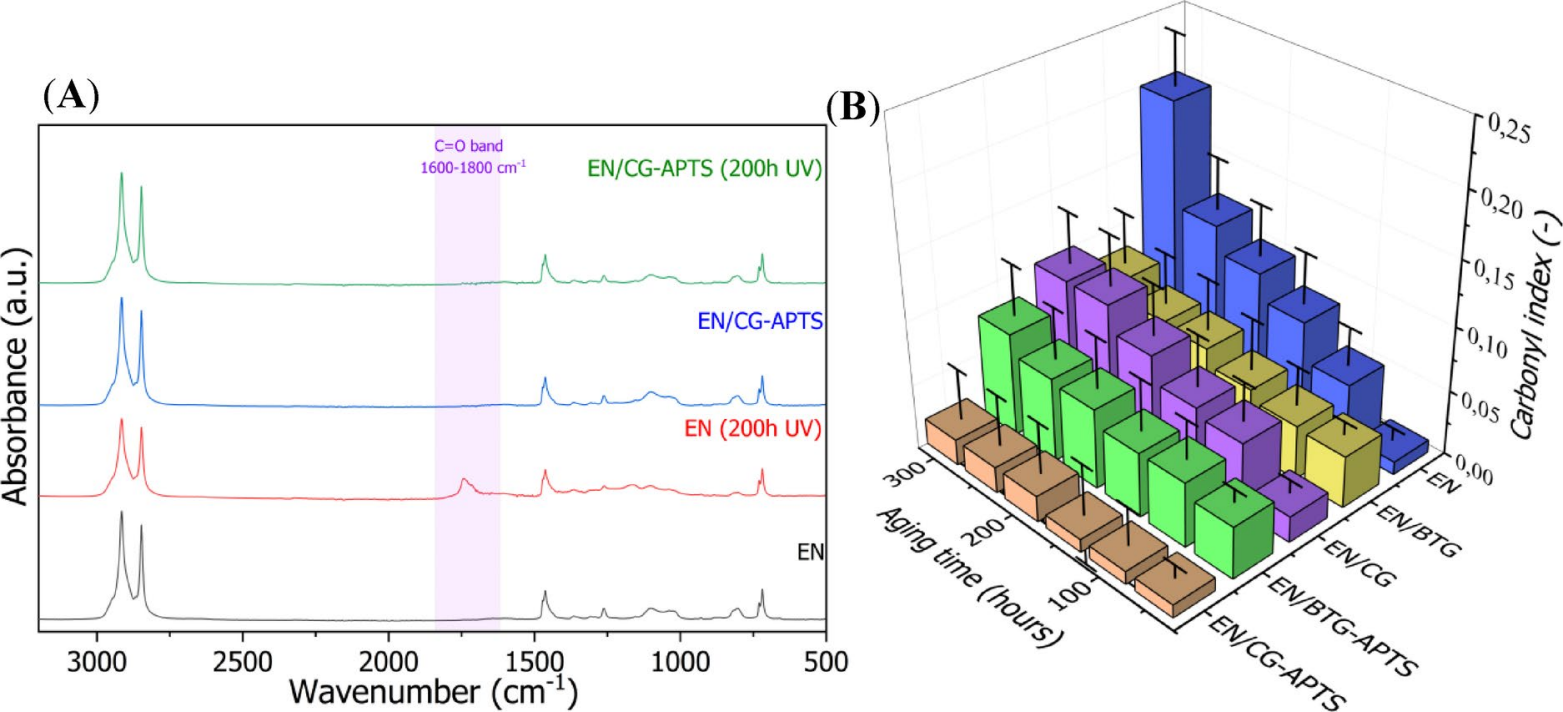
FTIR spectra (**A**) and carbonyl index (CI) (**B**) values measured for EN composites before and after UV aging.

The aging process of polymer materials occurs both within the macromolecular structure, but also on the surface. Surface changes in polymer materials exposed to aging factors typically involve surface oxidation, alterations in color, and variations in surface roughness. FTIR measurements were used to monitor the progress of surface oxidation in the EN polymer during UV aging, as indicated by the carbonyl index (CI). Figure 8 presents the FTIR spectra and the results of the carbonyl index (CI) for EN composites subjected to UV aging. It was observed that as the aging time increased, the intensity of the band at a wavelength of 1800–1680 cm^−1^, corresponding to the formation of C= O groups on the polymer surface, also increased. Consequently, the measured CI increased with aging time, reaching a value of 0.22 for pure EN after 300 h of UV aging. According to the literature, the CI values for this polymer after such aging time typically fall within the range of 0.2 to 0.3^44,45^. Importantly, the application of bio-fillers such as BTG and CG, as well as their silanized forms, resulted in a less drastic increase in CI during the aging process. For instance, after 300 h of UV aging, the CI values for EN/BTG and EN/CG composites were 0.09 and 0.11, respectively. While the CI values for the EN/BTG-APTS composite did not differ significantly from those of EN/BTG, silanization of CG resulted in a notable reduction in CI for the EN/CG-APTS composite, which remained below 0.02 throughout the experiment. These results indicate that bio-fillers in the form of coffee and black tea grounds can effectively inhibit the surface oxidation of aged EN, with silanization further enhancing their anti-aging efficiency. In fact, it is well-established in the literature that both of those bio-fillers are rich in antioxidant compounds, such as tannins and flavonoids in coffee or catechins and flavonols in black tea^46,47^. The presence of these active compounds in CG and BTG may have been responsible for the increased oxidation resistance of the EN composite surfaces. Moreover, the improved dispersion of the CG particles inside EN matrix could additionally enhanced their anti-aging activity, as evidenced by lower CI values.

**Fig. 9:**
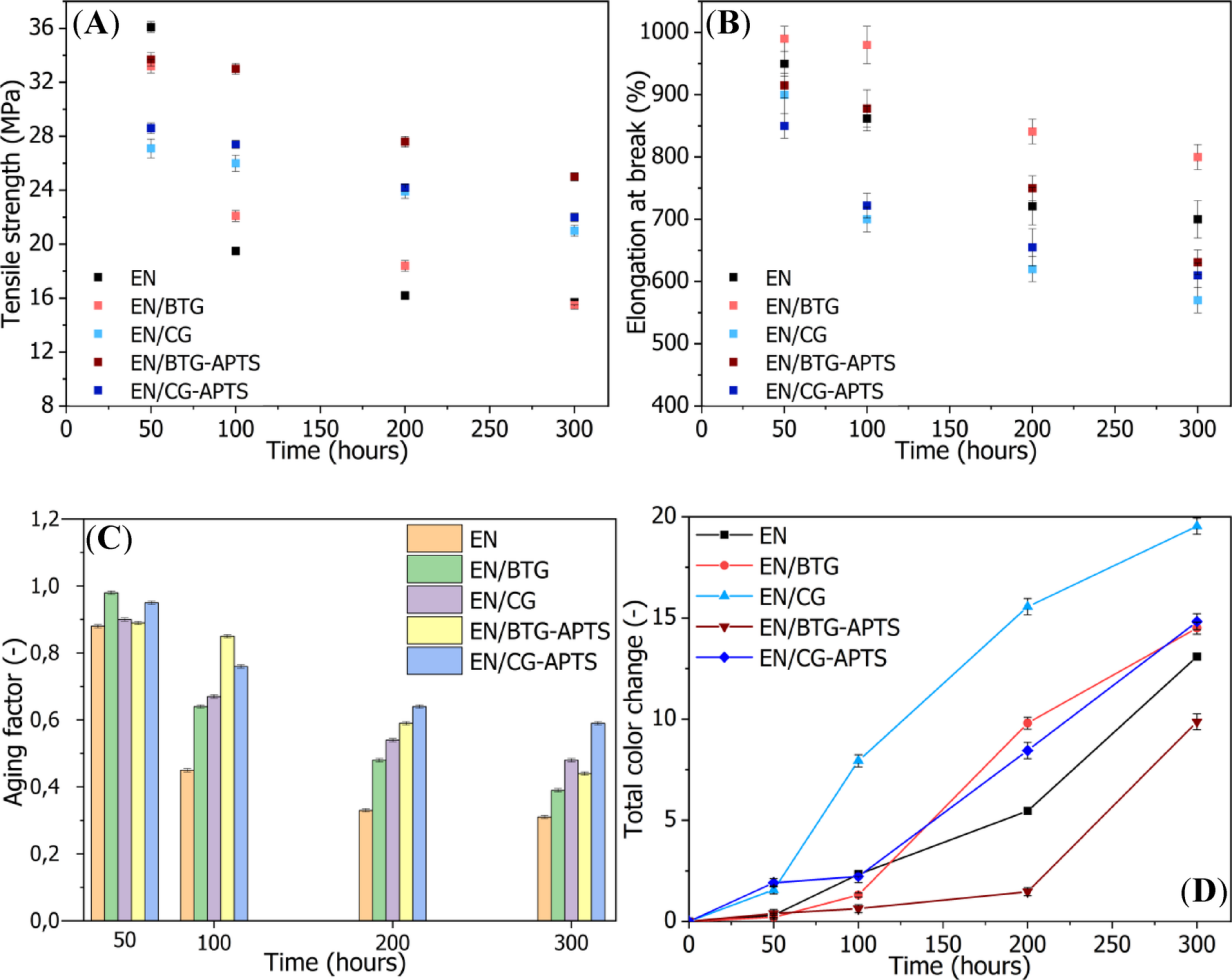
Tensile properties (**A**–**C**) and color change (**D**) determined for EN-filled composites subjected to UV aging.

From Figs. 9a and b, one sees that all EN composites subjected to UV-aging exhibited a decline tendency of tensile strength (T_S_) and elongation at break (E_B_). Especially, neat EN shown a dramatic decrease to 19.8, 17.0 and 16.5 MPa after 100, 200 and 300 h of UV aging, respectively. As the UV aging process progressed, a gradual reduction in the elongation at break of the reference EN material was also observed, decreasing from approximately 1000% to around 850% after 300 h of aging. This indicated an increase in the stiffness and brittleness of the polymer. The incorporation of coffee and tea waste into polymer composites can influence their mechanical properties depending on factors such as filler content, particle size, and interfacial adhesion. It can be seen that the presence of CG and BTG in the EN matrix resulted in composites with reduced mechanical properties due to the poor dispersion of those bio-fillers. One sees that the incorporation of CG as a bio-filler resulted in a more pronounced deterioration of the mechanical properties of EN compared to BTG. This may be attributed to the distinct particle structure and compatibility of these bio-fillers with the polymer matrix. SEM analysis revealed that CG exhibits a more irregular but dense structure, which may lead to poorer dispersion within the polymer and a higher tendency to concentrate local stresses. Yu et al.^48^ also reported a significant reduction in tensile strength, from approximately 50 MPa to 41 MPa, in PLA bio-composites filled with 5 wt% of spent coffee grounds. In other work, Marques et al.^23^ showed reduction in tensile strength of polypropylene (PP) composites by 7 MPa after application of 20 wt% of CG. This decrease in the tensile strength of CG-filled polymer composites was explained by poor adhesion between the CG natural fibres and the polymer matrix. Further SEM analysis of the composites revealed many gaps between bio-filler and matrix which hampered the proper load transfer between the PP and CG. The discussed studies, along with the results obtained here, indicate that the issue of compatibility challenges with CG or BTG bio-fillers remains relevant across various polymer matrices. Coming back to photostability aspect, EN composites filled with raw and silanized bio-fillers also exhibited a declining trend over the course of UV aging. However, the reduction in T_S_ and E_B_ for EN composites containing both untreated and silanized bio-fillers was significantly less pronounced compared to the pure EN material. This was reflected in the high values of the aging factor for these materials (Fig. 9c), with the following trend observed after 300 h of aging: EN < EN/BTG < EN/BTG-APTS < EN/CG < EN/CG-APTS. These results indicate enhanced UV resistance of EN composites filled with bio-fillers, particularly after functionalization using aminosilane.

UV aging of the EN composites also resulted in a gradual change in the color of the tested samples. As shown in Fig. 9d, all the studied composites exhibited significant color changes (∆E > 3) after 200 h of aging, except for the EN/BTG-APTS composite, for which the color change (∆E) was only 1.5. Initially, the filled EN composites exhibited a dark brown color. However, with prolonged UV aging, they gradually underwent discoloration, resulting in a noticeable reduction in color intensity. Even the unfilled EN copolymer exhibited ∆E values of 5.3 and 13.1 after 200 and 300 h of UV aging, respectively, which was attributed to the yellowing effect of the polymer. This is a typical phenomenon associated with the progressive oxidation process, leading to changes in the polymer structure, such as the formation of conjugated double bonds (C= C) or new chromophores containing conjugated groups^49^. The color change parameter results revealed that EN composites containing BTG exhibit less significant color change under prolonged UV exposure compared to those incorporating coffee grounds CG, which can be explained by the differences in their chemical composition and UV-absorbing compounds. Generally, black tea contains higher levels of polyphenols and tannins, which possess antioxidant properties and can mitigate photodegradation by scavenging free radicals generated during UV exposure. Additionally, the pigment composition in tea grounds is more stable under UV radiation compared to the darker, more oxidation-prone melanoidins present in coffee grounds. As a result, BTG-based composites maintain their original color more effectively, while CG-based composites undergo more pronounced discoloration due to the degradation of coffee-derived organic compounds. Furthermore, the silanization treatment with APTS most probably improved the interfacial adhesion between the BTG and the polymer matrix, leading to a more uniform dispersion of the filler and reducing the formation of surface defects that could accelerate UV-induced degradation. Additionally, the presence of silane molecules may have contributed to a protective effect by limiting the direct exposure of organic compounds to UV radiation, thereby further minimizing color changes in the composite material. These findings are consistent with the FTIR measurements, which indicated an increase in the number of carbonyl groups in the EN copolymer during UV aging. The most pronounced color change due to UV aging was observed in the EN/CG composite, which underwent severe discoloration after just 100 h of aging, with a ∆E value of approximately 7.5. Comparing the samples before and after silanization, it can be concluded that the silane treatment contributed to a slower rate of color change in composites containing silanized bio-fillers. These color observations highlight the protective effect of silane treatment on CG and BTG bio-fillers against photodegradation. Cichosz et al.^50^ also observed the positive effect of filler silanization using aminosilane on the UV-aging resistance of EN composites. The study suggested that the amino moieties present in the silane structure may have acted as free radical scavengers, thereby extending the shelf-life time of the EN-based composites.

## Conclusions

This study verified the possibility of using bio-waste products from the food industry, such as coffee and black tea grounds, as functional bio-fillers and/or coloring compounds for EN composites. It has been showed that the incorporation of CG and BTG waste bio-fillers into the EN matrix can serve as an effective strategy for producing low-cost polymer composites with a brown color characteristic. However, challenges remain, including the non-uniform dispersion of the fillers and a reduction in the mechanical properties of the resulting polymer materials. The presence of 5 wt% of CG and BTG bio-fillers resulted in reduction of tensile strength of EN by around 9 MPa and 3 MPa, respectively. Therefore, this work aimed to enhance the adhesion of these bio-fillers to the polymer matrix through functionalization with a silane coupling agent. The research demonstrated that silane treatment of CG and BTG using 3-aminopropyltriethoxysilane (APTS) enhanced the compatibility between the bio-fillers and the polymer matrix. This modification resulted in the improved filler dispersion, color uniformity of the composites and more effective stabilization against the detrimental effects of UV aging. Indeed, EN/CG-APTS and EN/BTG-APTS composites exhibited higher aging factors and lower ΔE and CI values under UV aging conditions compared to their non-modified counterparts. This work reveals that application of silanized coffee and black tea grounds as functional bio-fillers in polymer composites presents a highly attractive, low-cost, and sustainable approach to repurposing these bio-waste materials within polymer technology.

## Data Availability

The datasets used and/or analysed during the current study available from the corresponding author on reasonable request.
